# Preliminary Investigation of *Linum usitatissimum* Mucilage-Based Hydrogel as Possible Substitute to Synthetic Polymer-Based Hydrogels for Sustained Release Oral Drug Delivery

**DOI:** 10.3390/gels8030170

**Published:** 2022-03-09

**Authors:** Arshad Mahmood, Alia Erum, Sophia Mumtaz, Ume Ruqia Tulain, Nadia Shamshad Malik, Mohammed S. Alqahtani

**Affiliations:** 1 College of Pharmacy, Al Ain University, Abu Dhabi campus, Abu Dhabi 51133, United Arab Emirates; arshad.mahmood@aau.ac.ae; 2 AAU Health and Biomedical Research Center, Al Ain University, Abu Dhabi 51133, United Arab Emirates; 3 Faculty of Pharmacy, College of Pharmacy, University of Sargodha, Sargodha 40100, Pakistan; dr.sophia786@gmail.com (S.M.); umeruqia_tulain@yahoo.com (U.R.T.); 4 Faculty of Pharmacy, Capital University of Science and Technology, Islamabad 44000, Pakistan; nadia.malik@cust.edu.pk; 5 Nanobiotechnology Unit, Department of Pharmaceutics, College of Pharmacy, King Saud University, Riyadh 11362, Saudi Arabia; msaalqahtani@ksu.edu.sa

**Keywords:** *Linum usitatissimum* mucilage, hydrogel, nicorandil, copolymer, acrylamide

## Abstract

The aim of this study was to investigate the potential of *Linum usitatissimum* mucilage, a natural polymer, in developing a sustained release hydrogel for orally delivered drugs that require frequent dosing. For this purpose, nicorandil (a model drug)-loaded hydrogels with various feed ratios of *Linum usitatissimum* mucilage, acrylamide (monomer) and methylene bis-acrylamide (crosslinker) were prepared. The newly synthesized hydrogel formulations were probed fundamentally with respect to swelling behaviour, solvent penetration, and the release of the drug from the hydrogels. Later, the selected formulations were further characterized by Fourier-transform infrared spectroscopy, thermal analysis, X-ray diffraction analysis, and scanning electron microscopy. The swelling coefficient demonstrated a linear relation with the polymer ratio; however, an inverse behaviour in the case of monomer and crosslinker was observed. The drug release studies, performed at pH 1.2 and 4.5 and considering the dynamic environment of GIT, demonstrated that all formulations followed the Korsmeyer–Peppas model, displaying a slow drug release via diffusion and polymer erosion. FTIR analysis confirmed the successful grafting of acrylamide on linseed mucilage. Furthermore, scanning electron microscopy revealed a clear surface morphology with folds and pinholes in the hydrogel. Therefore, based upon the in-vitro outcomes, it can be concluded that a promising sustained release hydrogel can be prepared from natural polymer, *Linum usitatissimum* mucilage, offering many-fold benefits over the conventional synthetic polymers for oral delivery of drugs.

## 1. Introduction

The oral route of drug delivery is the most convenient way of administration but, at the same time, is associated with fluctuations in plasma drug concentrations that can be critical in certain diseases, for instance, cardiovascular problems [1]. In order to maintain a relatively uniform level of plasma concentrations, especially for the drugs with shorter half-life, and to avoid the problems of frequent administration, sustained release delivery systems have been the cream of the crop. Based on one mechanism or the other, a number of sustained release drug delivery systems have been established, including liposomes, microspheres, nano-emulsions and hydrogels, to name a few [2].

Hydrogels stand at the middle of the podium when it comes specifically to sustaining release delivery because of their ability to provide spatial and temporal control over the release of drugs. The development of tuneable hydrogels such as in-situ forming, stimuli (pH, temperature and enzymes) responsive [3,4,5], fatigue resistant [6,7], mechanical tune-ability with nano-particulate crosslinkers [8] and the degradation controlled hydrogel matrices are mainly based upon their physicochemical properties [9]. Over the past few decades, studies on novel drug delivery systems in general, and hydrogels in particular, have been focused on the so-called biocompatible synthetic polymers, which have dominated the era because of their consistency and considerable purity. However, when it comes to the biocompatibility, the biodegradability, the safety, the complications of the synthetic processes and, ultimately, the cost of production, there is no match to natural polymers. It has, further, been reported in the literature that natural polymers extemporized formulations via grafting and that improvised graft co-polymerization results in superabsorbent hydrogels with a swelling rate that ranges from a fraction of a minute to hours [10,11]. Considering the mentioned advantages, more and more natural polymers are being investigated in developing novel drug delivery systems under the umbrella of ‘naturapolyceutics’ for the delivery of all sorts of drugs.

Among the natural polymers, *Linum usitatissimum* (linseed) mucilage (polysaccharide) possesses the thickening, swelling and adhesive properties that render their potential for several applications in pharmaceutical preparations [12,13,14]. In the past decade or so, its initial utilization was reported as a gelling adhesive agent in combination with other polymers for buccal delivery [15,16] and colon targeting [17]. The swelling properties of the mucilage led to an investigation of their hydro-gelling potential; however, because of the lower mechanical strength, they were being used as an admixture with other polymers [18,19]. In our opinion, the graft copolymerization technique can help in fabricating harder and denser networks with enhanced mechanical strength of hydrogel with linseed mucilage.

Therefore, the aim of the study was to develop graft copolymeric hydrogel by free radical polymerization, using *Linum usitatissimum* (linseed) mucilage as a polymer with acrylamide monomer and methylene bis-acrylamide as a crosslinker for the gel frame. The composition based upon its intended characteristics was evaluated for influence on swelling, penetration of solvent and release of nicorandil, a model drug that has a very short half-life and that is an ideal candidate for the sustained release delivery.

## 2. Results & Discussion

### 2.1. Characterization of Linseed-Co-AAM Graft Copolymeric Hydrogels

**Figure 1:**
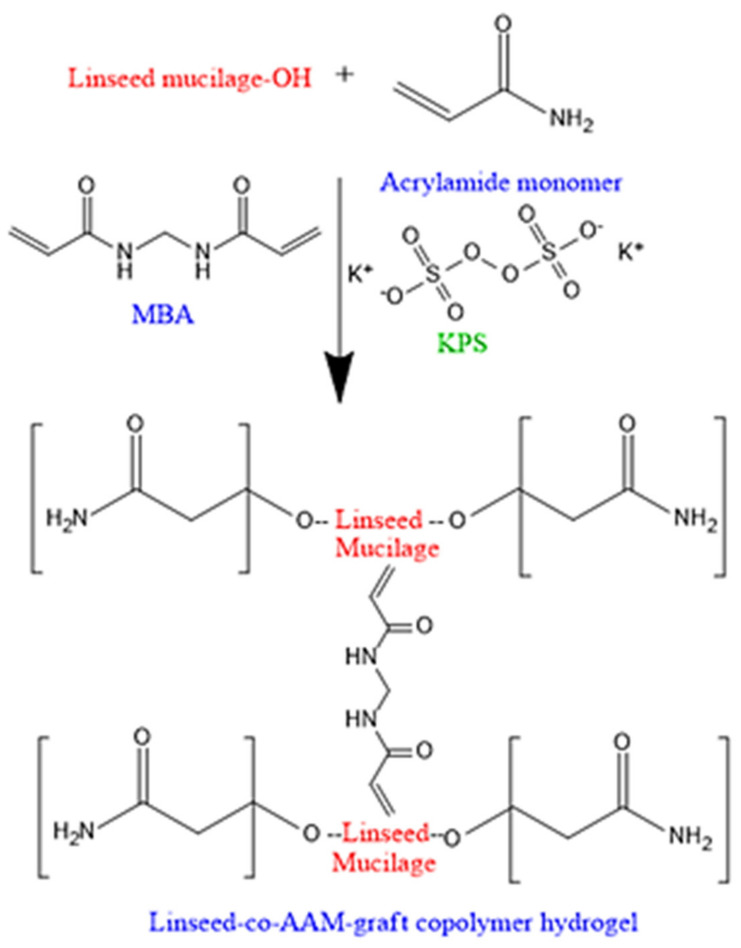
Schematic diagram for synthesis of linseed-co-AAM graft copolymeric hydrogels.

The mucilage contains water-soluble non-starch polysaccharides, rhamnogalacturonan and arabinoxylans are the main polysaccharides and these components of linseed, having hydroxyl groups attached with its backbone. The mucilage has an insufficient amount of hydrolytic stability that can be improved by introducing acrylamides with groups such as alkyl and hydroxyl alkyl. The proposed chemical structure of graft copolymeric hydrogel is illustrated in Figure 1.

#### 2.1.1. Swelling Studies

The swelling of naturally occurring polysaccharides depends upon the presence of hydrophilic/hydrophobic groups, degree of crosslinking, and elasticity of network [20]. Considering the physiochemical properties of the model drug, nicorandilnicorandil (weakly acidic drug with a pKa of 3.12), the swelling studies were performed at a pH of 1.2 and 4.5, which mimic the acidic environment of the gastrointestinal track, as per USP.

##### Effect of Varying Concentration of Monomer on Swelling Behaviour of Hydrogel

**Figure 2:**
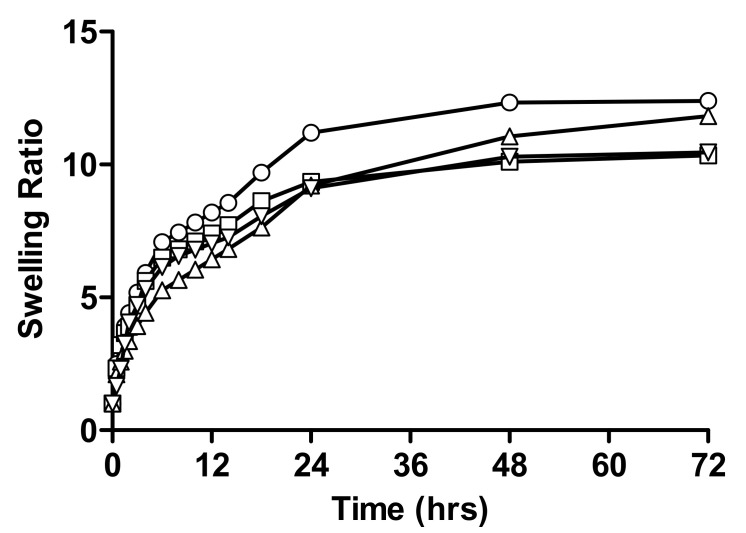
Relative swelling ratios of linseed-co-AAM graft copolymer hydrogel formulations with varying monomer concentration; (○) F1 at pH 1.2, (□) F2 at pH 1.2, (△) F1 at pH 4.5 and (▽) F2 at pH 4.5.

Comparative swelling ratios of different formulations of linseed-co-AAM hydrogel with varying monomer concentrations are shown in Figure 2. A slight decrease in swelling ratio was seen with increasing AAM concentration, as shown by swelling ratios 12.33 and 10.34 in the case of pH 1.2 and 11.82 and 10.56 in the case of pH 4.5 for F1 and F2, respectively.

According to the literature, hydrogels formulated with lower total monomer concentration revealed a higher swelling, and the equilibrium mass swelling of the hydrogels decreased with increasing total monomer concentration. For the constant gel volume, the decrease in the total monomer concentration results in an increase in the degree of dilution of the matrix in the constant gel, which also results in an increase in the equilibrium water content of the gel [21,22,23].

##### Effect of Varying Concentration of Linseed Mucilage on Swelling Behaviour of Hydrogel

**Figure 3:**
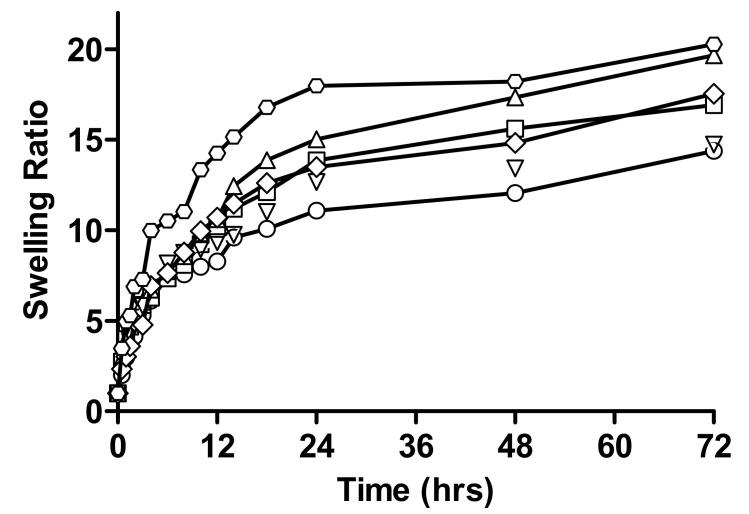
Relative swelling ratios of linseed-co-AAM graft copolymer hydrogels with varying linseed mucilage concentration; (○) F3 at pH 1.2, (□) F4 at pH 1.2, (△) F5 at pH 1.2, (▽) F3 at pH 4.5, (◇) F4 at pH 4.5 and (⬡) F5 at pH 4.5.

Comparative swelling ratios of different formulations of linseed-co-AAM hydrogel with varying mucilage concentration at pH 1.2 and 4.5 are specified in Figure 3. The swelling ratio increases with an increase in linseed mucilage concentration for F3, F4, and F5 as shown by swelling ratios 14.39, 16.92, 19.66, at pH 1.2 and 14.74, 17.54, and 20.27 at pH 4.5, respectively.

The swelling ratio of the hydrogels depends on the chemical structure of mucilage. Hydrogels that have hydrophilic groups swell greater then hydrogels containing hydrophobic groups because these groups break down in the presence of water, thus, minimizing their interaction with the water molecule [24,25]. The swelling of the polymer also depends on the number of hydrophilic groups on the polymeric network and the crosslinking density of the fine structure of the polymer. As the concentration of mucilage increased, the concentration of hydrophilic group increased having less crosslinking density which results in increased swelling of linseed mucilage [15,26].

##### Effect of Varying Concentration of Crosslinker

**Figure 4:**
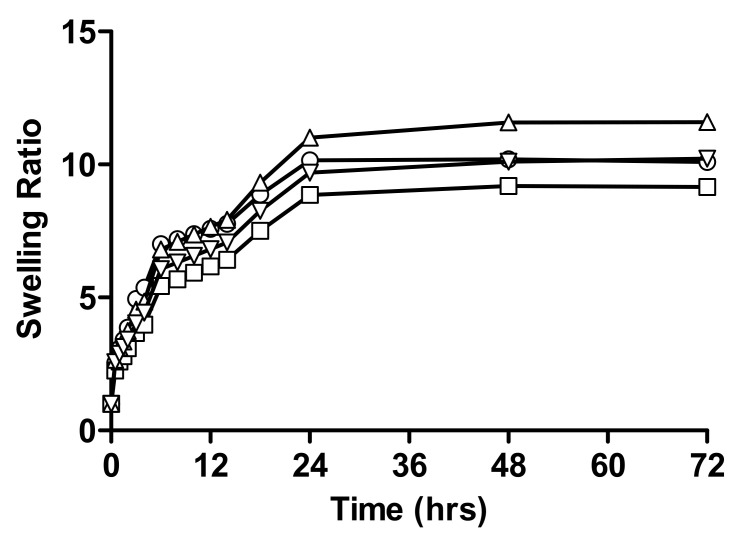
Relative swelling ratios of linseed-co-AAM graft copolymer hydrogels with varying crosslinker concentration; (○) F6 at pH 1.2, (□) F7 at pH 1.2, (△) F6 at pH 4.5 and (▽) F7 at pH 4.5.

Comparative swelling ratios of different formulations of hydrogels with varying crosslinker concentrations are shown in Figure 4. Results showed that, as the concentration of crosslinker increased, a decrease in swelling ratio was observed, which was 10.09, 9.16 at pH 1.2 and 11.59, 10.22 swelling ratios at pH 4.5. The consequence of a high concentration of crosslinker (N,N-MBA) in formulation having a higher crosslink density profile, thus, decreasing water absorbency of hydrogel [10]. It was estimated that the concentration of MBA had a substantial effect on the permeability, absorbency, and swelling features of hydrogels. The swelling was decreased by increasing concentration of MBA in acidic and buffer medium due to tighter structure and hindered mobility. Mechanical strength was improved, but the porosity of hydrogels was decreased; due to this, rate of drug release by diffusion was also decreased [27].

The literature indicates that power-law performances of swelling that increase the crosslinker lower the water absorbency. Higher crosslinking results in an increased degree of crosslinking in the polymer network, which results in low swelling [28,29].

#### 2.1.2. Percent Equilibrium Swelling (%ES) of All Formulations of Linseed-Co-AAM

The swelling index was carried out at pH 1.2 and 4.5 for all the above-mentioned formulations. Lower percentage of equilibrium swelling was obtained by increasing the crosslinker and monomer content in the hydrogel. With an increase in mucilage concentration, percentage of equilibrium swelling was increased [30].

Percentage of equilibrium swelling (% ES) of all formulations of hydrogels with varying mucilage, crosslinker and monomer concentrations at pH 1.2 and 7.4 are specified in Table 1.

### 2.2. Determination of Drug Loading

Quantity of drug integrated in different formulations of linseed-co-AAM is given in Table 2. Results indicate that, as the concentration of crosslinker increased, drug loading decreased from 40 to 30 mg, while, in the case of increased linseed mucilage concentration, drug loading increased from 58 to 66 mg. With an increase in monomer concentration, drug loading also decreased from 69 to 65 mg. The literature also indicates that, as the concentration of crosslinker was increased, crosslinking density was high as a result of decreased drug loading [27]. By the increase in monomer concentration, drug loading was decreased because dilution of the polymeric network increased in the AAM-based hydrogels [23]. By increasing linseed mucilage concentration, low crosslinking density of the polymer would occur due to an increase in hydrophilic groups of the polymer, which results in increased drug loading.

### 2.3. Instrumental Analysis

#### 2.3.1. Fourier Transforms Infrared (FTIR) Analysis

FTIR analysis was done to confirm AAM grafting on linseed mucilage. Secondly, to find out the loaded drug in the final formulation, discs revealed neither degradation nor reaction with the formulation. FTIR is based on the opinion that the basic components of a constituent that has chemical bonds usually show excitation and absorb infrared light at a specific peak frequency [31,32].

**Figure 5:**
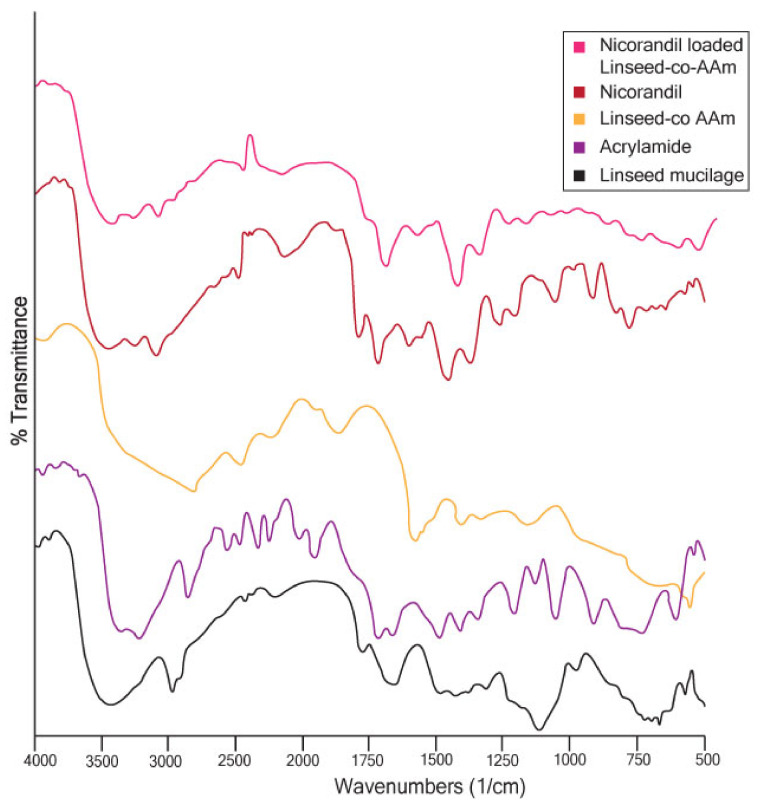
FTIR spectrum of linseed mucilage, AAM, linseed-co-AAM, Nicorandil and Nicorandil loaded Linseed-co-AAM formulation.

In regards to linseed mucilage FTIR, in Figure 5, the spectra showed 3433 cm^−1^ (OH stretching), 2870 cm^−1^ (aliphatic CH stretching) and 1722 cm^−1^ (carboxylic acid C=O stretching), which was probably due to acidic fractions present in linseed mucilage [33]. In the same overlay in the AAM spectrum, a sharp absorption peak at 1666.50 cm^−1^ indicates carbonyl C=O stretching [34]. In the IR spectrum of extra pure AAM, broadband at the region between 3300–3000 cm^−1^ indicates –NH stretching of the AAM unit. Carbonyl moiety of the AAM unit is found at the peak at 1615 cm^−1^ [35], while the spectrum of linseed-co-AAM with a clear and a sharp peak at 2938 cm^−1^ (C-H stretching) and a quite low-intensity peak at 3518 cm^−1^ (C-O stretching) is at 1042. The N-H intense peak at 1605 cm^−1^, the presence of NH and C=O vibrations confirm the presence of grafting of AAM with linseed mucilage [36].

The FT-IR spectrum of nicorandil is shown in Figure 5 and the following characteristic peaks were observed: the peak at 3235 cm^−1^ represents NH bending, the absorption band at 1663 cm^−1^ for (C=O, CONH) bending and peak at 1391 cm^−1^ for CH2 and 1597 cm^−1^ for the Pyridinium ring [37].

In nicorandil, the loaded linseed-co-AAM spectra absorption peaks at 1661 cm^−1^ (aromatic C=C bending) and 1389 cm^−1^ (aromatic C-H bending) confirmed that the drug did not show any chemical interaction with the hydrogel preparation. By recording the FTIR, the spectra of the drug confirmed that the model drug was evenly distributed within hydrogels. The FTIR spectra of the crosslinker, the monomer, and the polymer were also recorded to determine the uniform distribution of all these ingredients in linseed-based hydrogel [38].

#### 2.3.2. Scanning Electron Microscopy (SEM)

**Figure 6:**
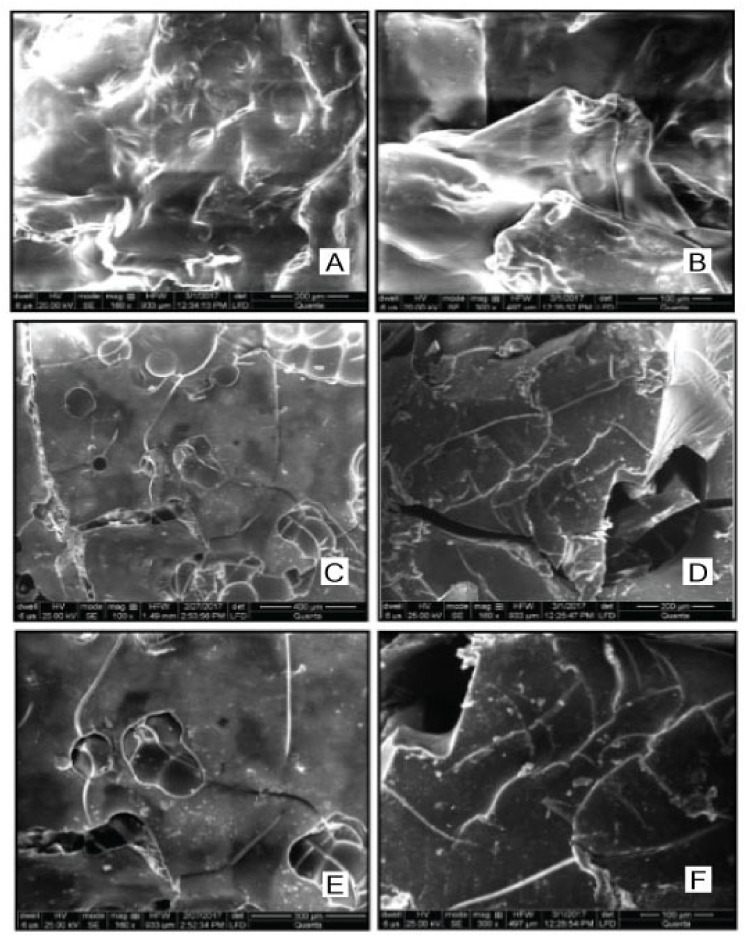
SEM image of F1 linseed-co-AAM formulation hydrogel with 100 µm (**A**) and 200 µm (**B**), F3 hydrogel with 300 µm (**C**) and 400 µm (**D**), F7 hydrogel with 100 µm (**E**) and 200 µm (**F**) scale.

For evaluating morphological features of prepared hydrogels, scanning electron microscopy was performed on grafted linseed-co-AAM hydrogels. Clear surface morphology was detected with folds and pinholes. As the concentration of AAM increased, folds and holes were denser [35,36]. In formulations F1, F3 and F7, as the concentration of AAM increased, folds and holes were more compact, as shown in Figure 6.

### 2.4. In-Vitro Drug Release Measurement

#### 2.4.1. In-Vitro Drug Release of Linseed-Co-AAM Formulations

**Figure 7:**
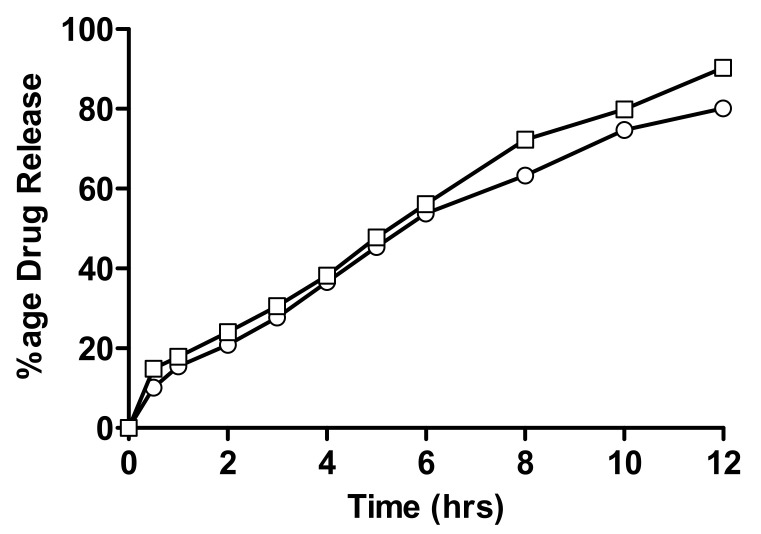
Percentage release of nicorandil from formulations having varying monomer concentrations; (○) F1 and (□) F2.

**Figure 8:**
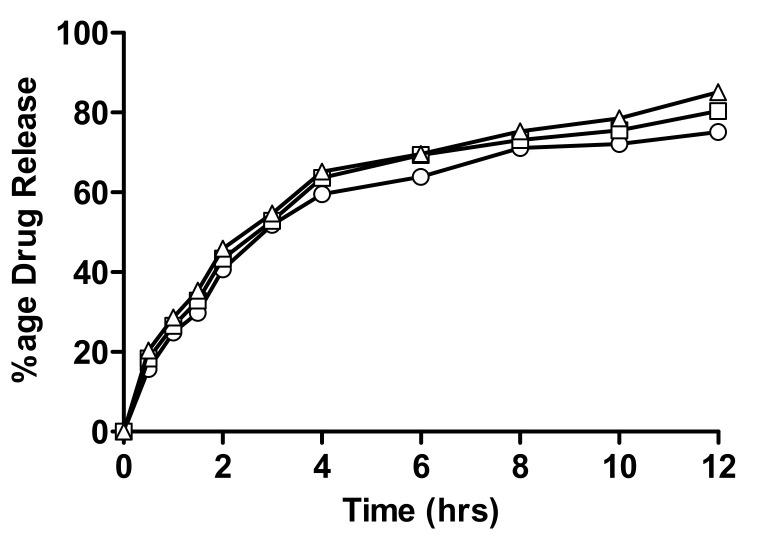
Percentage release of nicorandil from formulations having varying linseed mucilage concentration; (○) F1, (□) F2 and (△) F3.

**Figure 9:**
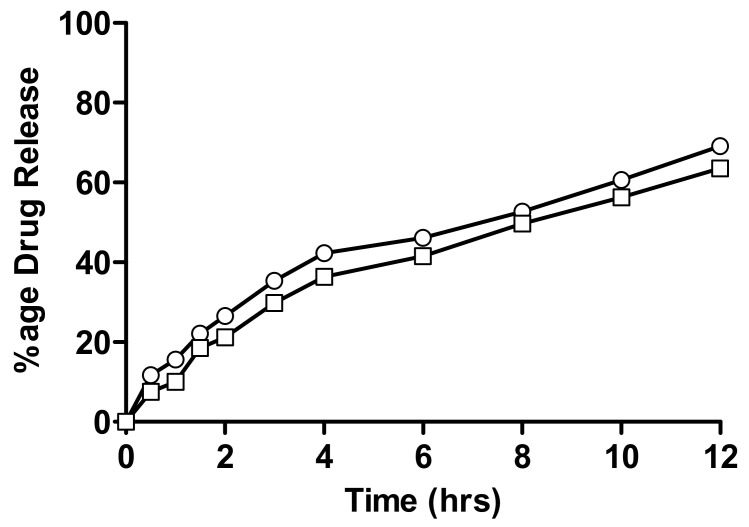
Percentage release of nicorandil with varying crosslinker concentration; (○) F6 and (□) F7.

The in-vitro drug release of linseed-co-AAM hydrogels with a varying monomer, linseed mucilage and crosslinker ratio is given in Figure 7, Figure 8 and Figure 9. Drug release from a hydrogel depends on the swelling, the interaction between drug and polymer behaviour, and the solubility of the drug [39]. Formulations with varying AAM concentrations showed an increase in drug release with decreasing AAM content. As for F1 and F2, the percentage of drug release after 12 h was 80.1% and 90.3%, respectively [39].

Formulations with varying contents of linseed mucilage (F3, F4 and F5) show higher release of nicorandil, i.e., 75.1, 80.4 and 85.1, respectively, as shown in Figure 8. This significant difference in drug release from both polymeric networks is due to higher swelling ratios of F5, as drug release is directly proportional to the swelling of the polymeric network. When water penetrates the gel network, swelling occurs and drug dissolution starts inside the network, followed by its gradual diffusion out of the polymeric matrix. Linseed mucilage is a hydrophilic polymer. Hydrogel porosity was also enhanced by high polymer concentration, which is attributed to the reduced crosslinking density that resulted in improved swelling [40,41].

As for F6 and F7, the percentage of nicorandil release after 12 h was 69.1% and 63.5%, respectively. Formulations with varying crosslinker concentrations showed a decrease in drug release with increasing crosslinker content because swelling and the interaction of the drug molecule with the physiological medium decreased, so drug release also decreased.

#### 2.4.2. Evaluation of Drug Release Kinetics

By using DD Solver software assessment of the drug release kinetics of numerous kinetic models, zero order, first order, Higuchi, Hixson–Crowell, and Korsmeyer–Peppas models were applied. The effects of different concentrations of crosslinker, monomer, and linseed mucilage concentrations on the kinetics of drug release from all linseed-co-AAM hydrogel formulations can be observed by the correlation coefficient (R2) values and the release rates mentioned in Table 3.

From the values of R2 of the line graph, it can be seen that the Korsmeyer–Peppas model best fits all formulations. This model is applied to determine the release mechanism of drugs, i.e., Fickian diffusion or non-Fickian diffusion. If the value of (n) for cylindrical hydrogel discs equals 0.45, it corresponds to the Fickian diffusion, whereas, when the (n) value is between 0.45 and 1.0, it represents the non-Fickian diffusion. Values of (n) for F6, F7, F1 & F2 preparations in this study were less than 1 and greater than 0.45, showing non-Fickian diffusion, while F3, F4 & F5 shows Fickian diffusion, as the (n) value is equal to 0.45 [42].

Accordingly, formulation F5 exhibited comparatively higher drug release due to greater swelling ratios. A decrease in the crosslinking density provides a porous network that aids the influx of water, followed by swelling. Drug release characteristics were found to be directly proportional to the swelling capacity and inversely proportional to crosslinker and monomer concentration.

## 3. Conclusions

Within the current study, hydrogels based on *Linum ussitatissimum* mucilage were successfully designed, with the ability to demonstrate a sustaining mechanism for drug delivery. Through a thorough analysis, it was observed that an increase in the monomer and crosslinker concentration resulted in a decrease in the swelling, as well as the rate and extent of drug release. However, increasing the mucilage concentration had the opposite impact, i.e., swelling and an increase in the rate and extent of drug release. Moreover, linseed-Co-AAM hydrogel depicts pH-independent swelling and drug release behavior. With particular emphasis on the sustained release delivery systems, formulations F2 and F5 were considered to be superior to other formulations; a deeper analysis involving in-vivo investigation is highly recommended.

## 4. Materials and Methods

### 4.1. Materials

Seeds of *Linum usitatissimum* were purchased from the local food market of Sargodha, Pakistan. Acrylamide, potassium persulfate, sodium hydroxide, and potassium dihydrogen phosphate were from Sigma Aldrich, Germany. N,N-Methylene bis-acrylamide was obtained from Fluka, Switzerland. Hydrochloric acid, absolute ethanol, and n-hexane were gained by Riedel-de Haen, Germany. Nicorandil was obtained as a gift sample from GETZ Pharma Pakistan (Pvt) Ltd., Karachi, Pakistan.

### 4.2. Methods

#### 4.2.1. Linseed Mucilage Extraction

Following the careful screening process, 200 g of *Linum usitatissimum *seeds were soaked in 600 mL of purified water with mild stirring at 25 °C. After a period of 24 h, the soaked seeds were incubated in a dry heat oven at 80 °C for 30 min. Mucilage was extracted via vacuum filtration and was washed with n-hexane using a cotton cloth, whereby n-hexane passed out very easily along with impurities and the filtrate was obtained in the form of mucilage. The purified mucilage was transferred to petri dishes and dried in an oven at 60 °C; the obtained scales were ground until a fine powder was obtained [14].

#### 4.2.2. Preparation of Linseed-Based Hydrogels

As shown in Table 4, seven different formulations were articulated via a free radical polymerization method, with varying concentrations of polymer (linseed), crosslinker (methylene bis-AAM), monomer (AAM), and potassium persulfate as initiator [28,43].

Briefly, linseed mucilage powder was dissolved in distilled water with continuous stirring at 70 °C. Once dissolved, potassium persulfate solution (in water) was added to it and stirring was continued for another 10 min. Afterwards, crosslinker (N, N-Methylene bis-AAM) solution and monomer were added to polymer-initiator solution at room temperature; the final weight was adjusted using distilled water. The above mixture was incubated in a water bath at 50 °C for an hour, and then temperature was gradually increased up to 80 °C until transparent hydrogels were formed. Cylindrical hydrogels were cut into 0.5 cm diameter discs, washed in an ethanol-water solution to remove the unreacted contents and dried in an oven at 50 °C for 24 h. Dried discs were stored in airtight containers for further characterization of hydrogels [43].

#### 4.2.3. Characterization of Linseed-Co-AAM Graft Copolymeric Hydrogels

##### Swelling Studies

Swelling studies were performed using the primly weighed dry hydrogel discs. The dry hydrogel discs were immersed in medium (having pH 1.2 and 4.5) and were allowed to swell until swelling equilibrium. At predetermined time intervals, swollen hydrogels were taken out of the medium; weight was noted after removing the excess water with filter paper that, ultimately, was placed back in same media. The following equation was used to calculate swelling behaviour:

(1) $$\mathrm{Swelling}=\frac{\mathrm{Ws}-\mathrm{Wd}}{\mathrm{Wd}}\;$$

where Ws = weight of hydrogel in swollen form, W_d_ = weight of hydrogel in dry form [20,36,44].

##### Percentage of Equilibrium Swelling/Equilibrium Water Content

The swelling was continued until each gel achieved a constant weight. Percentage of equilibrium swelling (%ES) or equilibrium water content (EWC) was determined by the following equation:

(2) $$\%\;\mathrm{ES}=\frac{\mathrm{Meq}-\mathrm{Wd}}{\mathrm{Meq}}\times 100$$

where Meq is the weight of swollen gel at equilibrium and W_d_ is the weight of dried gel discs [45].

##### Drug Loading

Nicorandil was used as a model drug for the development of sustained-release formulations for the treatment of hypertension [46]. Drug loading into the hydrogel disc was performed by using the adsorption method. A 1% w/v drug solution was prepared in phosphate buffer having pH 4.5. One disc of each formulation was dipped in 100 mL of 1% drug solution until swelling equilibrium. Discs were removed from the solution and washed out with distilled water to remove an excess of the drug. After, they were allowed to air dry at room temperature first and then oven-dried at 40 °C. The amount of drug loaded in the discs was determined by the following formula given in equation 3.

Total drug loaded = W_L_ − W_U_ (3)

where W_L_ = weight of dried drug loaded disc, W_U_ = weight of dried unloaded disc [46,47].

#### 4.2.4. Instrumental Analysis

##### Fourier Transform Infrared (FTIR) Analysis

The Fourier transform infrared (FTIR) spectra were recorded on an FTIR (prestige-21 Shimadzu) spectrometer for the pure model drug, unloaded hydrogel and drug-loaded hydrogel in order to identify formation of any new bond [48]. For this purpose, samples of the hydrogels and drug were mixed with KBr solution, dried, crushed and kept under hydraulic pressure (150 kg/cm^2^) to make the disc; spectra were recorded at a wavelength of 4000–500 cm^−1^.

##### Scanning Electron Microscopy (SEM) Analysis

SEM is a widely applied technique for the evaluation of shape and surface morphology of hydrogels. Dried hydrogel discs were cut to specific sizes and were fixed on an aluminium stub. Hydrogels were freeze-dried and then coated with gold in a high vacuum evaporator. The coated samples were scanned and examined under an electron microscope to expose surface morphology [49,50].

#### 4.2.5. In-Vitro Drug Release Study

In-vitro drug release studies were performed using USP-dissolution apparatus II at 37 ± 0.5 °C to evaluate the release behavior of all hydrogel formulations. Every disc was placed in dissolution medium, maintained at a temperature of 37 °C and stirred at a rate of 50 rpm to maintain a uniform drug concentration in the medium. An aliquot of 5 mL was withdrawn at specified time points, i.e., 0.5, 1, 2, 3, 4, 5, 6, 8, 10 and 12 h and absorbance of nicorandil was measured at a wavelength of 262 nm. In order to keep the dissolution medium volume constant, samples were replaced with an equal volume of fresh buffer maintained at 37 ± 0.5 °C. Standard calibration curves of nicorandil were obtained and absorbance was taken at 262 nm. Release kinetics of nicorandil from hydrogels was evaluated by dissolution data modeling by using DD Solver software [51].

Percentage of drug release in hydrogels was determined by using the following equation:

In vitro percentage drug release = F_t_/F_load_ × 100 (4)

where F_t_ = release of drug at time t, F_load_ = amount of drug loaded in disc.

### 4.3. Mathematical Models of Drug Release Kinetics

To evaluate the release pattern of nicorandil, and zero-order, first order, Higuchi, Hixson–Crowell, Korsmeyer–Peppas kinetic models were applied.

## Figures and Tables

**Table 1:** Percentage of equilibrium swelling of AAM-based hydrogels at pH 1.2 and 4.5.

Hydrogel Code	% Equilibrium Swelling (% ES)	
	pH 1.2	pH 4.5
F1	91.89	91.54
F2	90.26	90.53
F3	93.50	93.21
F4	93.72	93.26
F5	94.29	94.88
F6	90.09	91.37
F7	89.08	90.22

**Table 2:** Loaded drug in different formulations of linseed-co-AAM.

Hydrogel Code	Nicorandil-Loaded
	mg ± S.E.M
F1	69 ± 1.4
F2	65 ± 1.7
F3	58 ± 1.1
F4	60 ± 1.3
F5	66 ± 1.5
F6	40 ± 2.1
F7	30 ± 1.9

**Table 3:** Impact of various constituents on drug release from linseed-co-AAM graft copolymer.

Hydrogel Code	Zero Order Model		First Order Model		Higuchi Model		Korsmeyer–Peppas Model			Hixson–Crowell Model	
	R^2^	K_O_	R^2^	K_1_	R^2^	kH	R^2^	kKP	N	R^2^	kHC
F1	0.825	4.310	0.968	0.087	0.988	17.894	0.991	15.913	0.54	0.954	0.024
F2	0.824	3.928	0.976	0.074	0.992	16.329	0.995	14.541	0.54	0.952	0.020
F3	0.377	4.171	0.848	0.100	0.924	17.988	0.979	26.002	0.45	0.752	0.027
F4	0.284	4.288	0.828	0.111	0.898	18.578	0.975	28.427	0.45	0.724	0.030
F5	0.287	4.477	0.849	0.123	0.906	19.374	0.986	29.821	0.45	0.757	0.033
F6	0.707	3.385	0.899	0.058	0.994	14.243	0.996	15.641	0.46	0.852	0.016
F7	0.752	3.174	0.916	0.052	0.986	13.307	0.986	13.318	0.50	0.875	0.015

**Table 4:** Composition of numerous formulations of linseed-co-AAM graft copolymer.

Formulation Code	Linseed Mucilage	Acrylamide	Initiator	Crosslinker
	(g/100g)	(g/100g)	(g/100g)	(g/100g)
F1	1.0	12.5	0.2	0.2
F2	1.0	17.5	0.2	0.2
F3	1.0	15	0.2	0.2
F4	1.5	15	0.2	0.2
F5	2.0	15	0.2	0.2
F6	1.0	15	0.2	0.3
F7	1.0	15	0.2	0.4

## Data Availability

Not applicable.
